# Flexor Digitorum Longus Transfer in Chronic Plantar Plate Tears: Two Case Reports and Literature Review

**DOI:** 10.3390/reports7040087

**Published:** 2024-10-29

**Authors:** Antonio Córdoba-Fernández, Rocío Mateos-Carrasco, Antonio Jesús García-Gámez, Victoria Eugenia Córdoba-Jiménez

**Affiliations:** 1 Departamento de Podología, Universidad de Sevilla, Calle Avicena s/n, 41009 Sevilla, Spain; 2 Private Practice, Avenida Jose Manuel Caballero Bonald Edif. Solario, Local 6 y 7, 11405 Jerez de la Frontera, Spain; chiomarga81@gmail.com (R.M.-C.); piesportslp@gmail.com (A.J.G.-G.); 3 Private Practice, Calle Dr. Fleming 13, Bajo B, Castilleja de la Cuesta, 41950 Sevilla, Spain; luna.s__16@hotmail.com

**Keywords:** plantar plate tear, lesser toe deformity, metatarso-phalangeal instability, tendon transfer

## Abstract

**Background and Clinical Significance:** The plantar plate (PP) tear of the second metatarsophalangeal joint (MTPJ) is a common cause of forefoot pain in clinical practice. The PP is the main stabilizing structure of the joint and, together with the collateral ligaments, is the key to maintaining the stability of the MTPJ. Many surgical procedures have been described to repair PP tears. Currently, there is still controversy regarding which is the surgical superior option (direct versus indirect PP repair techniques). Transfer of the flexor digitorum longus tendon to the dorsum of the proximal phalanx is one of the surgical techniques described to treat PP tears associated with MTPJ instability. **Case Presentation:** We present two cases that developed instability of the second MTPJ secondary to chronic PP tear with symptoms resolved after transfer of the flexor digitorum longus (FDL). **Conclusions:** Currently, the literature review shows that the procedure seems to be the most consistent surgical option in chronic cases of PP tears.

## 1. Introduction and Clinical Significance

The plantar plate (PP) is a fibrocartilaginous structure that is key to maintaining the stability of the lesser metatarsophalangeal joints (MTPJs) together with the collateral ligaments. A PP tear in the second MTPJ is one of the most common causes of metatarsalgia in clinical practice. Chronic tear is the most common mode of PP injury, although rupture can also occur in acute trauma or in association with erosive rheumatoid arthritis [1,2]. In the early clinical stages, conservative treatment to reduce and stabilize the MTPJ may promote PP healing and reduce the risk of progression of deformity. However, patients often do not come to the clinic in the early stages of the disease, which often limits the role of conservative treatment. In severe cases that do not respond to conservative treatment, surgical treatment may be necessary.

The PP tear of the second MTPJ has been the subject of numerous investigations in the scientific literature; however, nowadays, there is still controversy about the best surgical approach to treat the pathology, especially in the second MTPJ. The common clinical presentation of the instability of the second MTPJ is characterized by chronic pain with positive drawer sing and mainly different degrees of deformity in the second toe. A very positive drawer test with increased vertical displacement and pain indicates a compromise in the integrity of the PP [3]. For decades, flexor tendon transfer has been considered the mainstay of treatment to stabilize the second subluxed MTPJ. However, in the past ten years, primary PP repair has been advocated as an alternative approach to pathology. Here, we present two cases of chronic PP tear involving the second MTPJ resolved by the transfer of flexor digitorum longus (FDL), metatarsal osteotomy, and proximal interphalangeal joint (PIPJ) arthrodesis. The results of the cases reported here, together with the available evidence, suggest that in chronic PP tears, indirect repair with transfer of the flexor digitorum longus tendon (FDL) associated or not with metatarsal osteotomy and PIPJ arthrodesis appears to be effective with clinical benefit and improved patient satisfaction [4].

## 2. Case Presentation

### 2.1. Case 1

A 64-year-old male presented with progressive metatarsalgia refractory to plantar orthosis after three years of treatment in the left foot. Physical examination of the second MTPJ showed little swelling, floating second toe, very positive drawer test, and fixed clawtoe without plantar hyperkeratosis.

**Figure 1:**
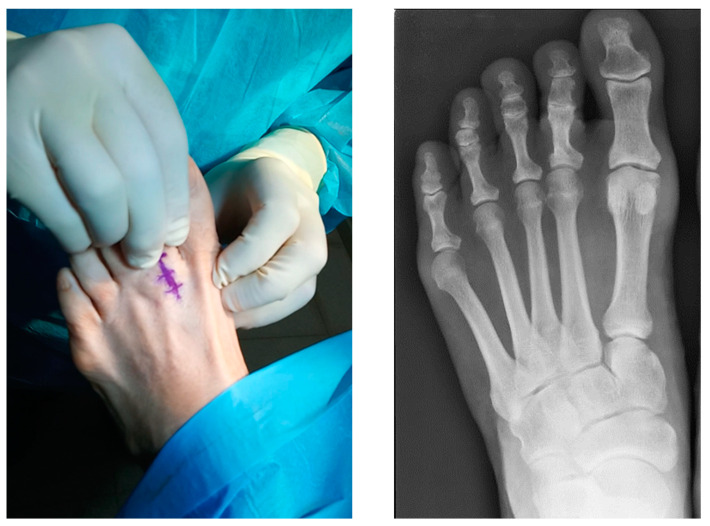
Clinical imagen where a positive drawer test can be observed (**left**). Radiological image showing the second subluxed MTPJ, dorsiflexion of the proximal phalanx and the degenerative PIPJ (**right**).

The weight-bearing anteroposterior radiographic examination showed a subluxed second MTPJ, dorsiflexion of the proximal phalanx, and degenerative PIPJ (Figure 1). In the longitudinal ultrasound image, the extensive PP tear visible through dynamic dorsiflexion maneuvers of the affected MTPJ could be visualized as a focal hypoechoic distal defect.

The proposed surgical procedure was flexor to extensor tendon transfer associated with metatarsal osteotomy and interphalangeal arthrodesis.

Using the dorsal approach, a curved S-shaped incision was made that extended from the MTPJ to the PIPJ. The PIPJ was identified, and the extensor tendon was transected and reflected proximally all the way to the MTPJ capsule. The collateral ligaments were then released, and the articular surfaces were minimally removed. Subsequently, the extensor tendon was removed from the Hood apparatus, and the MTPJ capsule was incised and released using a McGlamry elevator to reduce contracture.

**Figure 2:**
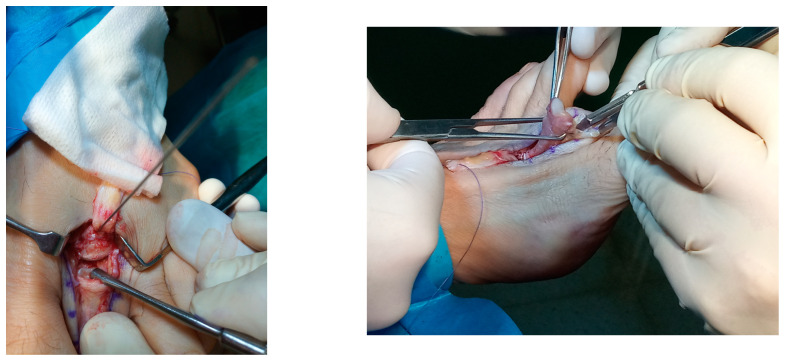
Image showing the fixation of the Weil osteotomy prior to the placement of the small and cannulated screw (**left**). Identification of FDL with release of the attachment of soft tissues and cut as distal as possible (**right**).

Using a sagittal saw, a second metatarsal Weil osteotomy was performed to decompress MTPJ and recompose the metatarsal parabola. The osteotomy was fixed in the desired position with one small and cannulated screw (QuickFix™, Arthrex, Munich, Germany). With the same approach and with plantarflexion of the ankle to relieve tension, using a curved hemostat, the FDL was identified and freed from the attachment of soft tissues, clamped and cut as distal as possible (Figure 2). The FDL tendon was divided into two through the midline raphe, and each half was held by a hemostat and passed in the medial and lateral aspects of the proximal phalanx.

**Figure 3:**
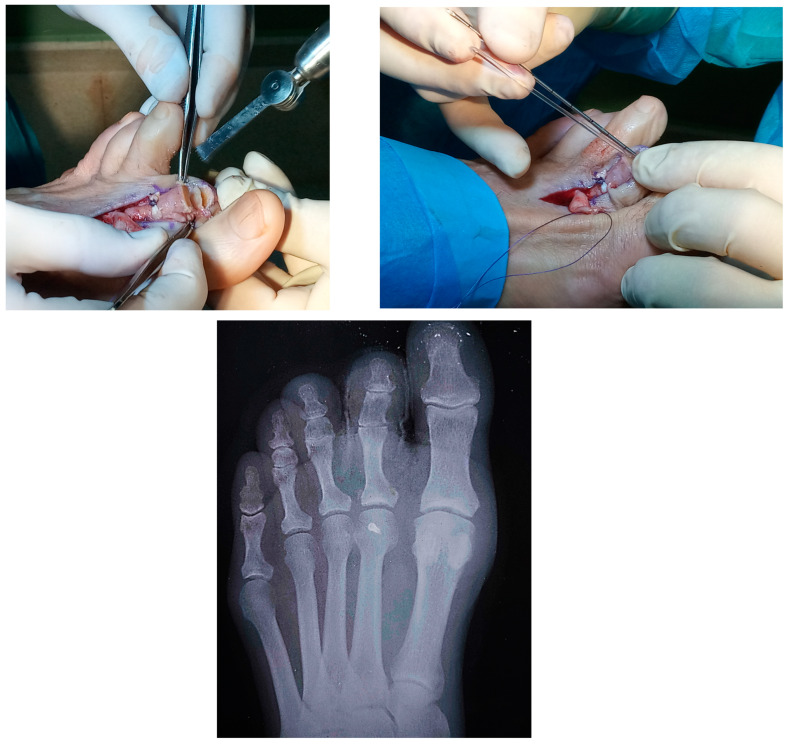
Image showing resection of the joint surfaces prior to performing end-to-end proximal arthrodesis (**left**). The image shows the measurement in the middle phalanx prior to insertion of the absorbable pin into the proximal phalanx to proceed with the PIPJ arthrodesis (**right**). Postoperative radiological image (**bottom**).

With the toe placed at 20 degrees of plantar flexion of the MTPJ, the tails of the FDL were dorsally crossed and sutured with the appropriate tension and the desired rest position slightly distal to the base of the proximal phalanx. Subsequently, end-to-end proximal arthrodesis of the PIPJ was performed using an absorbable pin of polylactic acid (Trim-It ^TM^, Arthrex, Munich, Germany). After resection of the PIPJ, a pilot hole was drilled with specific wire into the proximal phalanx. The middle phalanx was drilled and measured, their absorbable pin was then drilled into the proximal phalanx, and the other end was cut at the appropriate measured amount of pin for the middle phalanx. The pin was then gently bent with a hemostat, and the middle phalanx was placed over the pin. Finally, the extensor tendon was conveniently sutured, and after radiologic control, routine wound closure was performed (Figure 3). A postoperative compression dressing was applied, and the patient was discharged home on the postoperative day.

The foot was placed in a postoperative shoe with a rigid rocker bottom sole. A non-weight bearing status was advised for 2 weeks until the wounds healed, and the stitches were removed. From weeks 2 to 4, partial weight bearing on the heel was allowed. and the K-wires were removed in week four. From the fourth week, progress to full weight bearing was allowed and, from the sixth week, a return to normal shoes was allowed. Four months after surgery, the patient regained normal function with improvement in the stability of the affected MTPJ and absence of pain, continuing to be asymptomatic after three years of follow-up.

### 2.2. Case 2

**Figure 4:**
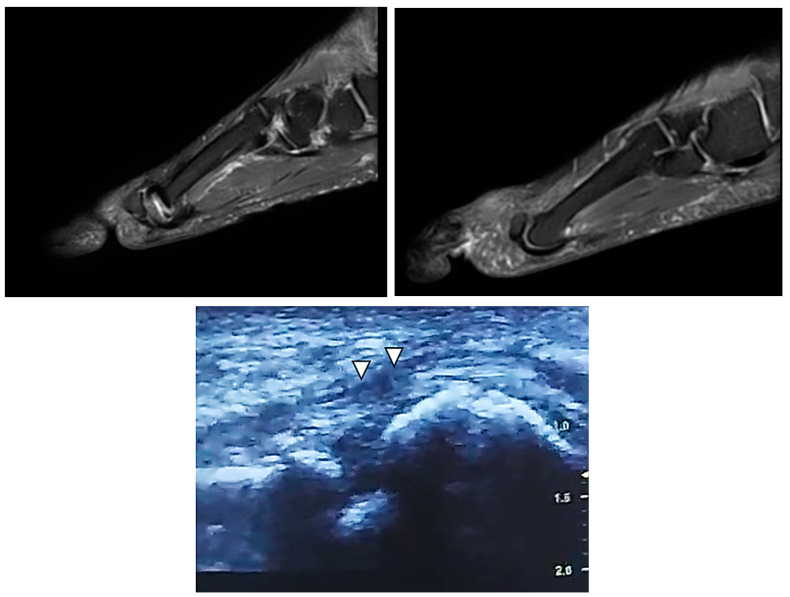
Diagnostic images corresponding to case 2. Second T2-weighted MTPJ MRI sequence with evidence of partial PP tearing (**left**). Normal MRI image corresponding to the healthy third MTPJ of the same foot (**right**). Longitudinal ultrasound image showing the full-thickness PP tear (arrows) visualized as a focal hypoechoic defect that extends through the distal substance from the articular to the plantar surface (**bottom**).

A 54-year-old male presented with progressive pain in the second MTPJ of the left foot refractory to conservative treatments (orthosis, physical therapy, and infiltration with steroids). The patient had undergone surgery a year earlier for metatarsalgia and hallux valgus consisting of chevron and Akin osteotomy in the first ray, and Weil osteotomy and PIPJ arthrodesis in the second MTPJ. The physical examination of the second MTPJ showed little swelling and a very positive drawer test. Dorsoplantar radiological examination showed MTPJ subluxed, and magnetic resonance imaging (MRI) reported postoperative changes in the MTPJ joints of the hallux and second toe with residual hallux valgus. The second MTPJ MRI showed thinning/elongation with signal changes in the T2-weighted sequences with evidence of partial PP tearing displaced laterally. No abnormalities were observed in the supporting structures of the joint or other related forefoot pathologies. The longitudinal ultrasound (US) image showed a full-thickness PP tear visualized as a focal hypoechoic defect that extends through the distal substance from the articular to the plantar surface (Figure 4).

**Figure 5:**
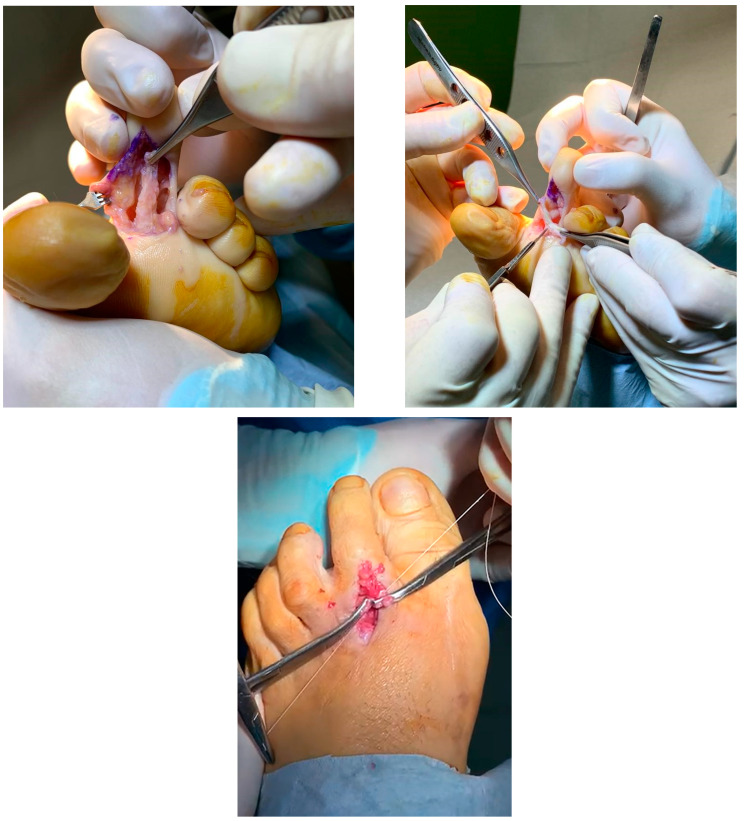
Image showing approach on the plantar aspect of the second toe with the FDL released from its insertion at the inferior border of the distal phalanx (**left**). Division of FDL tendon in two through raphe (**right**). Slips of FDL being held over the proximal phalanx and toe in the corrected position during suturing (**bottom**).

The surgical procedure consisting in flexor to extensor transference as described by Parrish was proposed [5]. A longitudinal dorsal incision was made from the MTPJ to the PIPJ. Subcutaneous tissues were reflected, avoiding neurovascular bundles medially and laterally. The dissection was taken to the extensor sheath, and the tendon and periosteum are split in line with the incision, exposing the dorsum of the proximal phalanx. A channel was created medially and laterally around the proximal phalanx with a periosteal elevator. With a longitudinal incision on the plantar aspect of the second toe, the flexor tendon was released from its insertion at the inferior border of the distal phalanx and divided into two through the midline raphe. Under appropriate tension, each half of the tendon was then transferred proximally and dorsally around the proximal phalanx and sutured together using a 3-0 non-absorbable suture (Figure 5). Finally, routine skin closure was performed, and a postoperative compression dressing was applied. The same postoperative management as in case 1 was carried out.

Five months after surgery, the patient regained normal function with an improvement in the stability of the affected MTPJ and absence of pain, continuing to be asymptomatic after two years of follow-up.

## 3. Discussion

The problem of lesser toe deformities, metatarsalgia, and their treatment remains controversial among scientific schools and clinical practitioners. A PP tear of the second MTPJ is a common cause of forefoot pain in clinical practice. Direct PP repairs, tendon transfer, and osteotomies techniques have been widely used, but combinations of these techniques are discussable in the literature.

When the PP is injured, the plantar flexion mechanism is altered with a consequent instability of the MTPJ and a decrease in the plantar flexion strength. This causes a relative imbalance in which the dorsiflexors gain mechanical advantage, accentuating any already existing dorsiflexion toe deformity. In acute PP tears, direct repair may be indicated; however, as in the presented cases, chronic PP tears can usually require balance around the affected lesser MTPJ associated with the treatment of pathological conditions such as hallux valgus to reduce forefoot overload and pressure under the affected lesser MTPJ. In a subluxed MTPJ associated with PP tears, a shortening metatarsal osteotomy can be indicated. Some studies suggest that an increase in the second metatarsal protrusion distance together with an increase in the intermetatarsal angle measured on radiographs may correlate with PP injuries [6,7]. However, the surgical approach to the pathology must also ensure biomechanical restoration of the unstable MPJ and must address the underlying etiology and dysfunction of the MTPJ that produce the condition. The index procedure, in fact, resembles the combination of the well-known parts, but the complex approach in these two cases demonstrated superior results to compare with the isolated procedures. The critical analysis of the available literature appears to indicate advantages in favor of the combined approach [8,9]. In case 2, although the patient had undergone hallux valgus and second clawtoe surgery with Weil osteotomy and PIPJ arthrodesis, it is very likely that the results of the procedure were insufficient to achieve the spontaneous healing of a chronic PP tear.

Currently, questions and debate remain about the healing capacity of the PP. Cadaver studies in the PP have demonstrated the presence of a vasculature network that extends from the surrounding soft tissues to the proximal and distal attachments. In the presence of a torn PP, there is increased vascular density suggesting that the PP is a structure that can attempt to heal [10]. In the same way, chronic PP tears are characterized by attenuation and the presence of degenerative fibrous tissue inside their central portion that can make healing difficult [11]. Clinical experience shows that when chronic PP tears occur, conservative treatment can eliminate symptoms and prevent progression of the deformity but cannot achieve MTPJ joint stability and realignment without surgical intervention.

Different classifications have been proposed to define the instability of the second MTPJ describing the clinical progression of the deformity. Signs and symptoms characterized the initial stage by pain and swelling of the MTPJ to a variable deformity of the toe that ranges from subluxed to a dislocated joint [12]. The positive drawer test, or “positive Lachman”, is the first objective sign of instability of the MTPJ [13]. It is a reproducible test based on five grades (0 = negative test to 4 = dislocated joint). As in both reported cases, the rigid grades III and IV are associated with important PP tears, the drawer test being very positive [14]. In both cases, the patients presented incapacitating clinical symptoms characteristic of chronic disease. However, often in severe grade IV, PP tears are usually associated with minimal symptoms with several fixed MTPJ deformities (dislocated MTPJ).

Coughlin et al. proposed a grading system of metatarsophalangeal PP tears that combined clinical findings and anatomical aspects. This classification correlates the location, shape, and size of the PP tear observed in cadaver specimens with the clinical signs and symptoms observed in patients with PP tears [3]. Other cadaver studies have allowed classification of the types and extent of PP tears associated with increased deformity of the second toe [15].

Although these classifications are of obvious academic interest, they may not always be precise or easy to establish clinically without the support of imaging probes. Physical examination is initially performed for diagnosis, but imaging can confirm clinical suspicion and play a key role in the management of pathology. High-resolution MRI or US can confirm the presence of PP degeneration and tear and exclude other pathologies. The available evidence shows that MRI is the best option to provide additional information on the supporting structures of the joint; however, dynamic US in the sagittal plane is more sensitive, suggesting that negative US virtually rules out a PP injury [16]. In case 2, the second MTPJ MRI showed thinning/elongation with signal changes in the T2-weighted sequences with evidence of important partial tearing being laterally displaced. In both presented cases, the US examination showed a characteristic appearance compatible with a partial tear of the PP characterized by a focal anechoic defect with loss of echogenic granular texture in the lateral portion of the PP suggesting a partial thickness tear [17,18].

Currently, the surgical approach for the PP tear of the second MTPJ includes a variety of direct or indirect repair techniques with or without industry-driven devices. In the last decade, many surgical techniques have been described to manage MTPJ deformities by means of PP repair, but only some of them have been applied, relating treatment with the severity of PP injuries. Clinical experience and prospective clinical studies show that in patters of PP injured grade 0 (attenuation) and grade 1 (little transverse distal tear), conservative treatments or soft tissue releases and joint decompression with or without shortening metatarsal osteotomies are effective with improved clinical results and may promote spontaneous healing of PP [8]. Although direct repair could be justified for an isolated acute event or in cases of grade II tears, in grade III and IV cases (chronic PP tears) such as those presented here, with evident instability in the MTPJ and the presence of degenerative fibrous tissue within the middle or distal portion of the PP, or grade IV with extensive PP damage, direct repair is not viable.

To date, available evidence shows that the most consistently successful salvage procedure is considered an indirect technique to re-establishing MTPJ instability with flexor to extensor tendon transfer with or without Weil osteotomy [19]. While surgical treatment should be individualized, available evidence shows that in grade IV PP tears, overall patient satisfaction levels remain high when performed with the appropriate indications and concomitantly with other procedures [9]. Transferring the FDL to the extensor tendon is considered an appropriate procedure for grade III and IV tears. However, in severe deformities of the lesser toes associated with chronic PP tears, such as those reported here, surgical treatment should be individualized and can require a sequential process with a combination of procedures to obtain adequate correction for deformity.

Taylor is the first author to be credited with performing a long and short flexor transfer into the extensor Hood for the correction of claw toes that Girdleston previously had described for flexible toe deformities associated with neuromuscular disorders [20]. Later, Parrish reported a modification of the original technique, transferring only the FDL and splitting it, passing each half of the tendon through each side of the proximal phalanx [5]. Most recently, other authors have modified this technique, and now there are various approaches with a single dorsal incision, two or three incisions, and FDL tenodesis to the proximal phalanx [21,22]. The approaches for specific tendon transfer depend on other procedures being concomitantly performed, previous procedures, or the severity and complexity of the deformity.

In case 1 reported here, the technique was performed with a single dorsal incision that allowed metatarsal osteotomy, tendon transposition, and arthrodesis to be performed simultaneously. However, in case 2, where only the tendon transposition was performed, the two-incision technique was considered adequate. The main complication reported in the literature with the technique is the risk of postoperative stiffness of the MTPJ [8,9,14]. To avoid this, the tendon should be transferred under physiological tension, and after performing the procedure, the position of the MTPJ should be evaluated and, where appropriate, a lengthening or extensor tenotomy should be performed. In the present manuscript, the authors do not appeal to scientific aims; nevertheless, the two reported cases combined with other procedures and with a long follow-up period are a clear example of the clinical advantages of the FDL transfer technique as a salvage procedure in chronic PP tears.

## 4. Conclusions

Although more rigorous clinical investigations are required to fully understand the effectiveness of direct versus indirect PP repair procedures in the management of lesser MTPJ instability, the available evidence shows that an indirect repair procedure consisting of the transfer of FDL associated with PIPJ arthrodesis with or without shortening metatarsal osteotomy provides a predictable and reproducible result for patients with rigid deformities and chronic PP tears as presented here.

## Data Availability

All data generated during this study are included in this article. The data for this study were obtained using the EMR to obtain the details needed to complete two case reports. To protect the identities of patients, this information is reserved.
